# Improved Shell Color Index for Chicken Eggs with Blue-green Shells Based on Machine Learning Analysis

**DOI:** 10.3390/foods14173027

**Published:** 2025-08-29

**Authors:** Huanhuan Wang, Yinghui Wei, Lei Zhang, Ying Ge, Hang Liu, Xuedong Zhang

**Affiliations:** Hangzhou Academy of Agricultural Sciences, Hangzhou 310024, China; weiyinghui0403@foxmail.com (Y.W.); ifuo@163.com (L.Z.); geying1122@126.com (Y.G.); lhomme@126.com (H.L.); bigzhengliang@hotmail.com (X.Z.)

**Keywords:** blue-green egg, L*a*b* values, visual classification, machine learning, shell color index

## Abstract

Shell color is a commercially valuable trait in eggs, and blue-green eggshells typically exhibit multiple color subtypes. To explore the relationship between the CIELab system and visual color classification and develop simplified discrimination indices, 2274 blue-green eggs across seven batches were selected. The L*, a*, and b* values of each egg were measured, and average visual classification (AveObs) was calculated from four numeric categories (Light = 1, Blue = 2, Green = 3, Olive = 4) separately assigned by four observers. After batch correction using ComBat, four algorithms—linear discriminant analysis (LDA), random forest (RF), support vector machine (SVM), and neural network (NNET)—were compared. Correction substantially reduced the coefficients of variation of the L*, a*, and b* values. Correlations emerged: L* and b* (−0.722), a* and b* (0.451), and L* and a* (−0.088), while correlations of the L*, a*, and b* values with AveObs were −0.713, 0.218, and 0.771, respectively. The LDA model achieved superior comprehensive performance across all data scenarios, with the highest accuracy and efficiency as compared to the SVM, NNET, and RF models. Among the LDA functions, LD1 explained 78.53% of the variance, with L*, a*, and b* coefficients of −0.134, 0.063, and 0.349, respectively (ratio ≈ 1:0.47:2.60). Simplified formulas based on the L*, a*, and b* values were constructed and compared to the existing indices C* (=$\sqrt{{a*}^{2}+{b*}^{2}}$) and SCI (=L* − a* − b*). The correlation between L* − 2b* and AveObs was −0.803, similar to those for C* (0.797) and SCI (−0.782), while the correlation between L* − 4C* and AveObs was −0.810, significantly higher than that for SCI (*p* < 0.05). In conclusion, the LDA model demonstrated optimal performance in predicting color classification, and L* − 4C* is an ideal index for grading of blue-green eggs.

## 1. Introduction

Eggshell color is a commercially significant trait that drives consumer choice; it not only protects egg contents against solar radiation [1,2] but is also closely correlated with shell strength and thickness [3,4]. Chemically, eggshell color is determined by the pigments protoporphyrin IX, biliverdin, and zinc chelates of biliverdin. Brown shells are primarily composed of protoporphyrin IX, while blue-green shells contain various proportions of biliverdin and protoporphyrin IX [5,6,7,8]. Although accounting for a relatively small market share, chicken eggs with blue-green shells are popular among consumers in certain countries, particularly in Southeast Asia, owing to their unique appearance and rich nutritional content [9,10]. Consumers typically prefer eggs with uniform shells, which can be improved by genetic selection [11,12,13]. However, prior studies have primarily focused on brown eggshells, while relatively few have examined blue-green eggshells. Non-uniformity is reportedly prevalent in blue-green eggshells, which is attributed to diverse chromatic classifications from light through shades of blue, green, and olive-green (also named yellow-green or green-brown) [14]. Green-brown shells contain elevated concentrations of both protoporphyrin IX and biliverdin, resulting in a yellowish hue as compared to green shells and a greenish hue as compared to brown shells [15]. The subjectivity of visual color classification can be avoided through comprehensive evaluation by multiple observers, which yields superior accuracy [16]. Although visual color assessment is the most direct approach, human classification is not only time-consuming and labor-intensive but also limited to broad chromatic categories due to the inability to discriminate subtle color differences below the perceptual threshold (ΔE < 3) [17,18]. Consequently, objective and efficient methods for quantifying eggshell color are critically needed.

The CIELab color system established by the International Commission on Illumination (CIE) has been widely adopted for color measurement due to its full coverage of the human-visible spectra, user-friendly operation, and device independence [19,20]. The CIELab color system quantifies chromatic attributes by measuring the L*, a*, and b* values, where L* represents lightness, ranging from 0 (black) to 100 (white); a* indicates red-green chromaticity, with positive and negative values denoting red and green, respectively; and b* indicates yellow-blue chromaticity, with positive and negative values denoting yellow and blue, respectively [21]. Two indices have been adopted to assess eggshell color—chroma (C*) and the shell color index (SCI)—derived from the simplified CIELab formulas C* = $\sqrt{{a*}^{2}+{b*}^{2}}$ and SCI = L* − a* − b*, respectively. The C* value has been applied to investigate the impact of blue eggs laid by pied flycatchers on mating behavior [22], while the SCI has been used to assess the breeding process of uniform dark-brown eggs [12]. Instrumental measurements, such as those with Minolta colorimeters, demonstrate generalizability across varying environments and different chicken breeds [12,16,20]. However, as shell coloration is fundamentally determined by sight, it remains unclear whether instrumental measurement is consistent with visual classification, whether visual inspection can be replaced by automated methods, and whether there are better indices of eggshell colors. At present, machine learning algorithms for classification include decision tree, discriminant analysis, support vector machine, neural network, k-nearest neighbor, and Bayesian methods, although the applicability and classification performance vary depending on the type of dataset [23,24,25]. Therefore, the aim of the present study was to assess variations in visual classification of blue-green eggs versus instrumental measurements to provide a scientific foundation for eggshell color analysis. The contributions include the following: (1) systematic algorithm comparison for optimal model selection; (2) a novel methodology of simplifying complex models to simple formulas; and (3) the development of a superior simplified index for practical applications.

## 2. Materials and Methods

### 2.1. Egg Samples

From 2020 to 2023, eggs laid by hens of a black-bone, blue-eggshell chicken line were collected, comprising seven batches totaling 2274 eggs (with a single representative egg selected from each laying hen in generations G5 to G8) as study samples (Table 1). The chicken line, designated “BG”, was bred from segregating progeny of crosses between Dongxiang blue eggshell chickens and Jiangshan black-bone chickens [26]. All rearing practices were approved by the Animal Ethics Committee of Hangzhou Academy of Agricultural Sciences (NKY-SEC-2020-004, 10 February 2020; NKY-SEC-2023-003, 8 March 2023) (Hangzhou, China).

### 2.2. Eggshell Color Measurement and Visual Classification

The shell color parameters (L*, a*, and b*) near the blunt end of each egg from each batch were promptly measured using a Minolta CR-400 colorimeter (Konica Minolta, Inc., Tokyo, Japan) with an 8 mm aperture, illuminant D65, and pulsed xenon lamp as a default light source. The instrument was calibrated with a standard white tile before each measurement. L* represents lightness (0 to 100, where 0 = black and 100 = white), a* reflects red-green chromaticity (−120 to +120, with lower negative values indicating greener hues and higher positive values indicating redder hues), and b* reflects yellow-blue chromaticity (−120 to +120, with lower negative values indicating bluer hues and higher positive values indicating yellower hues). The C* and SCI values of each egg were calculated in accordance with Equations (1) and (2), respectively:

(1) $$C*\;=\sqrt{{a*}^{2}+{b*}^{2}}$$

SCI = L* − a* − b*(2)

**Figure 1:**
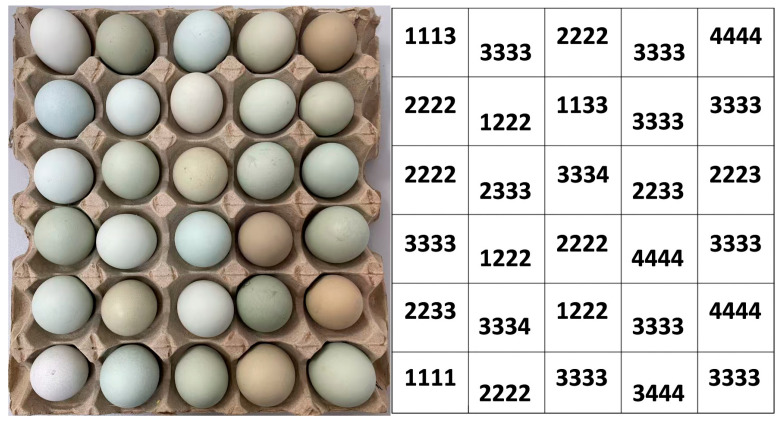
Images of observed eggs on a tray (left half of the figure) and the corresponding composite classifications (4-Obs) (right half of the figure).

In accordance with the methodology described in a previous study [16], four independent observers (Obs1, Obs2, Obs3, and Obs4) visually categorized the shell color of each egg into four classes: Light, Blue, Green, and Olive. Visual color assessment was performed in a specialized viewing room under illuminant D65 (6500 K correlated color temperature) using full-spectrum light emitting diode lamps. After assigning numerical values to the shell color classifications (Light = 1, Blue = 2, Green = 3, and Olive = 4), the individual assessments were combined to generate a composite visual color classification from all four observers (designated as 4-Obs). As an example, images of observed eggs on a tray and the composite classifications are shown in Figure 1. Subsequently, the average visual color classification (AveObs) for each egg was calculated using Equation (3):

AveObs = (Obs1 + Obs2 + Obs3 + Obs4) ÷ 4(3)

The whole dataset of eggshell color is provided in supplementary Table S1 (including the measured L*a*b* values, 4-Obs, and AveObs of each sample), and the statistical details of 4-Obs and AveObs are presented in Table 2. Here, “consistent samples” refer to those with 4-Obs values of 1111, 2222, 3333, or 4444, totaling 860 eggs, while the remaining 1414 eggs were categorized as “inconsistent samples”. AveObs comprises 13 distinct values spaced at intervals of 0.25 ranging from 1.00 to 4.00. Notably, 72.8% of the samples had AveObs values of 2.00 to 3.00.

### 2.3. Data Analyses

#### 2.3.1. Batch Correction

Batch correction of the L*, a*, and b* values across seven batches was performed using the ComBat function of the R 4.5.0 package “sva” (https://bioconductor.org/packages/release/bioc/html/sva.html, accessed on 6 May 2025). The corrected values of sample j in batch i were computed as Corr_X_ij_ = (X_ij_ − α_i_)/γ_i_, where α_i_ and γ_i_ are the estimated location and scale parameters for batch i, using the parametric empirical Bayes methods. AveObs was included as a covariate in the design matrix to preserve true biological variation from the influence of batch correction. Detailed R code for batch correction is provided in supplementary File S1. The coefficients of variation (CV) of the mean L*, a*, and b* values of the seven batches were calculated and compared before and after correction.

#### 2.3.2. Corrected Data Statistics

Statistical analysis of the corrected data was conducted using JMP Pro 14.0 software (https://www.jmp.com/zh-hans/home, accessed on 6 May 2025). The means, standard deviations, and CVs of the L*, a*, and b* values were calculated for all samples, along with the pairwise correlation coefficients and correlations with AveObs.

#### 2.3.3. Machine Learning Comparisons

Algorithm training was implemented using the train function of the R 4.5.0 package “caret” (https://topepo.github.io/caret/, accessed on 6 May 2025). For three data scenarios (all samples, consistent samples, and inconsistent samples), 70% of the data were randomly selected as the training set and the remaining 30% served as the testing set. Based on the characteristics of eggshell color data (e.g., multiple categories and continuous variables) and classification requirements (linear or non-linear approaches, computational efficiency, and robustness), four algorithms—linear discriminant analysis (LDA), random forest (RF), support vector machine (SVM) with a radial basis kernel, and neural network (NNET)—were trained to fit the L*, a*, and b* values to the AveObs value of the training set, with 10-fold cross-validation. LDA is suitable for multi-class problems with approximately normal distributed features. SVM with the radial basis kernel can handle non-linear classification boundaries. NNET can capture complex non-linear relationships. RF is an ensemble approach with robustness to overfitting. These four classical algorithms represent different methodological approaches and were selected to provide comprehensive comparison of classification performance. For RF, 500 trees were used with sqrt(p) variables sampled at each split. SVM employed a radial basis kernel with automatic scaling. NNET used a single hidden layer with 10 neurons and a sigmoid activation function. Detailed parameter specifications are provided in Supplementary Table S2. The trained models were used to predict shell color categories in the testing set, as compared to the actual observed classifications. The accuracy, precision, recall, F1-score, and kappa value of the models were evaluated using confusion matrices in accordance with the following equations:

Accuracy = Number of correctly classified samples (sum of diagonal elements in confusion matrix) ÷ Total number of samples in all classes(4)

Precision = Number of correctly classified samples in a specific class ÷ Total number of samples predicted as that class (sum of row)(5)

Recall = Number of correctly classified samples in a specific class ÷ Total number of actual samples in that class (sum of column)(6)

F1-Score = 2 × (Precision × Recall) ÷ (Precision + Recall)(7)

Kappa = (Accuracy − Pe) ÷ (1 − Pe), where Pe is the expected proportion of correct classifications by random chance(8)

Additionally, the robustness and efficiency of the four algorithms were evaluated using standardized metrics. For robustness assessment, noise sensitivity was quantified by adding independent Gaussian noise to the L*, a*, and b* values simultaneously at 10% of the standard deviation of each feature, with the mean prediction error rate (1 − Accuracy) over 30 repetitions serving as the metric. For computational efficiency comparison, training efficiency (samples processed per second during training) and prediction efficiency (samples classified per second during inference) were measured.

#### 2.3.4. Optimal Model Analysis and Simple Color Index Construction

Based on the comprehensive performance (e.g., accuracy and prediction efficiency) across the four algorithms, LDA was identified as the optimal model. The discriminant functions and coefficients of LDA were then analyzed to derive a simplified equation to determine the color index. The optimal coefficient (k value) for the formula was established by incremental testing at intervals of 0.1. Finally, Fisher’s Z-test was applied to compare the newly developed color index with existing indices (C* and SCI), and the ideal index was refined through optimization.

## 3. Results

### 3.1. Effects of Batch Correction

**Figure 2:**
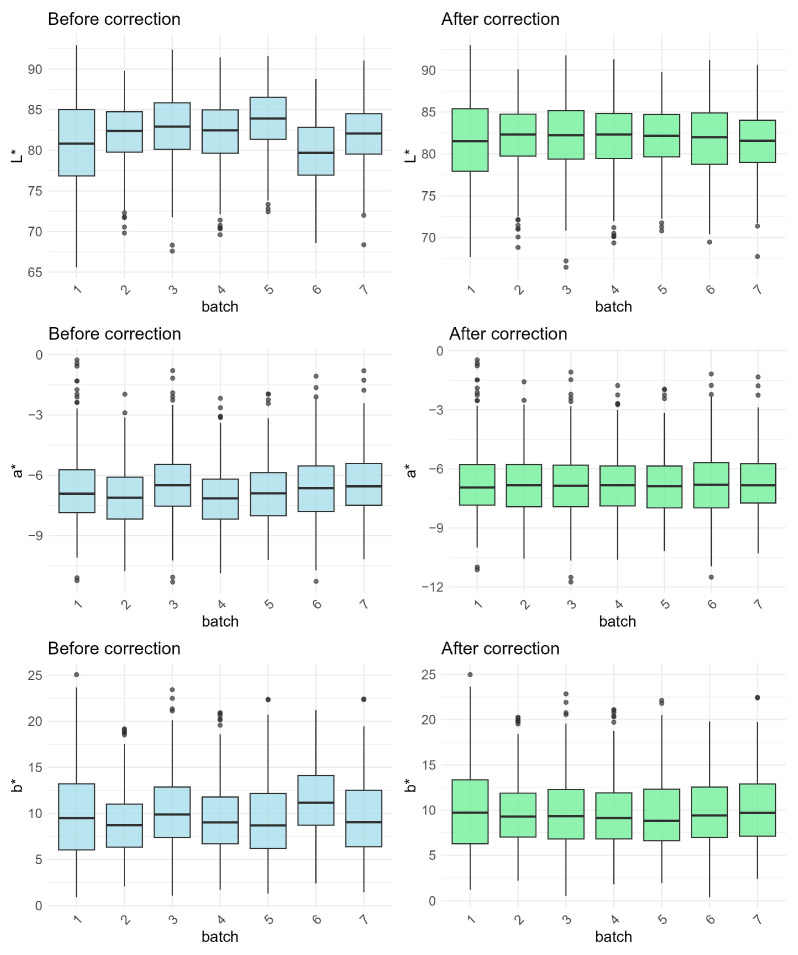
Distributions of the L*, a*, and b* values of seven batches before and after correction.

Box-and-whisker plots of the L*, a*, and b* values of all seven batches before and after batch correction are provided in Figure 2. As shown, the median lines of all seven batches became markedly aligned after correction for the L*, a*, or b* value. The CVs of the L*, a*, and b* values across all seven batches before and after correction are presented in Table 3. Prior to correction, the L*, a*, and b* values exhibited a relatively low, moderate, and high CV of 1.55%, 4.42%, and 8.40%, respectively. After correction, the CVs were significantly reduced, with reduction rates of 67.9% to 83.9%. These results indicate that batch correction with the ComBat function had substantially reduced inter-batch variability.

### 3.2. Statistical Results of Corrected Data

A statistical summary of all samples after correction is presented in Table 4. The CV of the L* value was relatively lower than that of the a* and b* values (5.19% vs. 24.51% and 40.84%, respectively), indicating substantial phenotypic variation within the samples. Almost no correlation was observed between the L* and a* values (−0.088), while there was a strong negative correlation between the L* and b* values (−0.722), and a moderate positive correlation between the a* and b* values (0.451). Moreover, the L* value was strongly negatively correlated with AveObs (−0.713), whereas the a* and b* values were weakly and strongly positively correlated (0.218 and 0.771), respectively. These results suggest that shell color classification is predominantly driven by the b* and L* values.

### 3.3. Classification Results of Machine Learning Algorithms

The main classification performance of the four machine learning algorithms (LDA, RF, SVM, and NNET) in three data scenarios is summarized in Table 5. For consistent samples, all models performed well, with accuracies exceeding 0.93, while LDA and the SVM achieved the highest accuracy of 0.941. In terms of F1-score and Kappa value, or noise sensitivity and prediction efficiency, LDA consistently demonstrated superior performance across most metrics. For inconsistent samples, all models showed lower accuracies below 0.45, with LDA performing the best (0.448). LDA also had the highest Kappa value (0.335) and prediction efficiency (140,334 samples/s), while SVM had the highest F1-score (0.482) and lowest noise sensitivity (0.095). For the full dataset, LDA remained the top-performing model with an accuracy of 0.450, followed by SVM, NNET, and RF, with accuracies ranging from 0.395 to 0.439. The Kappa value and prediction efficiency mirrored the accuracy trends, consistently favoring LDA, which achieved the highest Kappa value (0.357) and prediction efficiency (172,803 samples/s). Meanwhile, SVM had the highest F1-score (0.472), and NNET had the lowest noise sensitivity (0.261). These findings indicate that the LDA model exhibited the best comprehensive performance among the four algorithms for shell color classification, with SVM and NNET performing moderately and RF showing the poorest performance. The results of precision, recall, and training efficiency in Supplementary Table S3 further supported this conclusion, with LDA achieving the highest training efficiency (4807 samples/s for all samples) and superior recall values across three data scenarios. The confusion matrices of the four models are presented in Supplementary Table S4, where the actual and predicted classification numbers for each category revealed that misclassifications predominantly occurred between adjacent color categories.

### 3.4. Analyzed Results of the Optimal Model

According to the results derived from the LDA model, three discriminant functions (LD1, LD2, and LD3) had variance contributions of 78.53%, 21.05%, and 0.42%, respectively, indicating that LD1 alone accounted for most of the shell color variation, while LD2 contributed modestly and LD3 contributed minimally. The mathematical equations of the LD1, LD2, and LD3 functions are provided in Equations (9), (10), and (11), respectively:

LD1 = −0.134 × L* + 0.062 × a* + 0.349 × b*(9)

LD2 = 0.051 × L* + 0.834 × a* − 0.100 × b*(10)

LD3 = −0.362 × L* + 0.356 × a* − 0.392 × b*(11)

The coefficients of the L*, a*, and b* values in the primary discriminant function (LD1) were −0.134, 0.062, and 0.349, respectively, indicating that the b* and a* values exerted positive influences on shell color differentiation, while the L* value had a negative influence. Among these, the b* value exhibited the strongest impact, followed by the L* value. The ratio of the coefficients of the L*, a*, and b* values was approximately 1:0.47:2.60. Based on the signs and magnitudes of the coefficients in LD1, while referencing the current shell color indices C* (=$\sqrt{{a*}^{2}+{b*}^{2}}$) and SCI (=L* − a* − b*), the simplified equations L* − kb* (including the major two impact factors, i.e., L* and b*) and L* − kC* (including all the three colorimeter parameters, i.e., L*, a*, and b*) were derived for practical applications. Here, k denotes a coefficient with an exact value to be determined.

### 3.5. Optimized Results of Simple Color Indices

**Figure 3:**
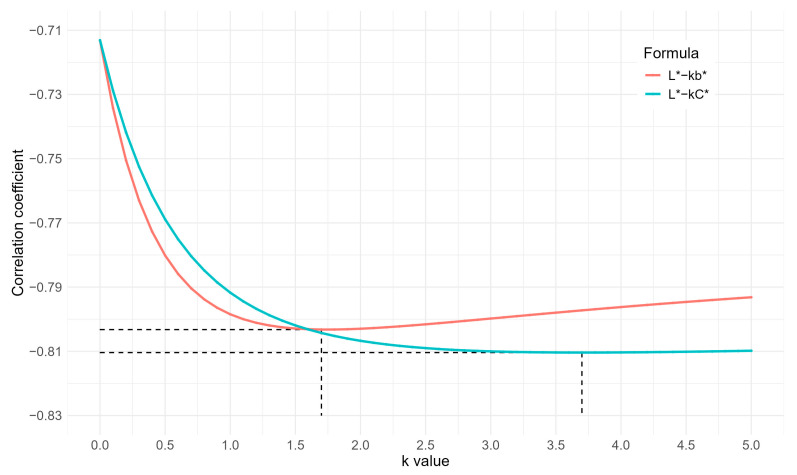
Curves of the correlation coefficient with the corresponding k value of two simplified formulas. Red and green curves show changes to the correlation coefficient between the formula and AveObs value, along with the k value in formulas L* − kb* and L* − kC*, respectively. Dashed lines represent the minimum negative coefficients (i.e., strongest correlations) and the corresponding k values.

The correlations between the simplified formulas (i.e., L* − kb* and L* − kC*) with AveObs with different k values are shown in Figure 3. When k = 1.7, the equation L* − kb* had the strongest correlation coefficient with AveObs (r = −0.803). Notably, at three decimal places for the correlation coefficients, the equation L* − 2b* (with k = 2, the integer closest to 1.7) achieved an identical r-value of −0.803. Similarly, for the equation L* − kC*, the strongest correlation coefficient (r = −0.810) occurred at k = 3.7 (the closest integer is 4). At k ≥ 3, L* − kC* yielded the same r-value of –0.810 (retaining three decimal places). Consequently, the simplest formulas L* − 2b* and L* − 4C* were selected for comparative analysis with the current shell color indices C* and SCI. Comparing to C*, L* − 4C* adds L* with weight −4 to C*. Similarly, comparing to SCI, L* − 2b* omits a* and weights b* by 2.

The correlations between SCI, C*, L* − 2b*, and L* − 4C* with AveObs, along with the correlation differences (*p*-values), are presented in Table 6. As shown, the absolute r-values of SCI and C* were both less than 0.8, indicating weaker correlations with AveObs as compared to L* − 2b* (−0.803) and L* − 4C* (−0.810). Notably, L* − 4C* demonstrated the strongest correlation with AveObs, with a statistically significant difference as compared to SCI (*p* = 0.010, <0.05), indicating the superior index.

### 3.6. Practical Distribution with Superior Color Index (L* − 4C*)

**Figure 4:**
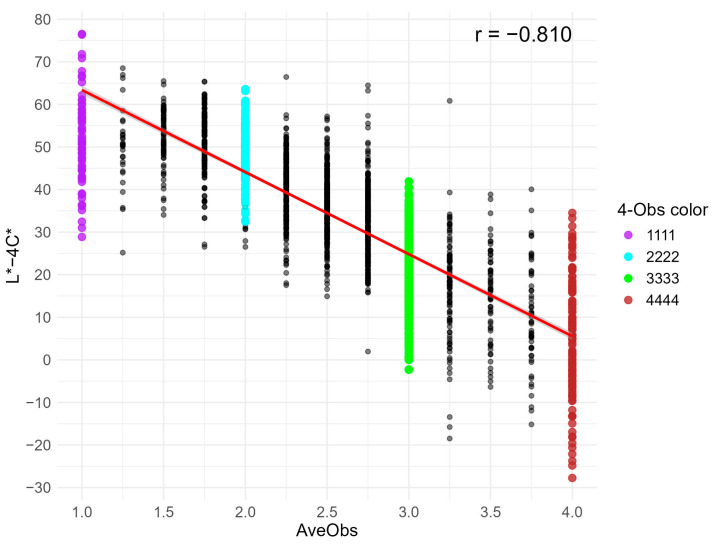
Scatter plot of AveObs and L* − 4C* values. Purple, blue, green, and brown points represent consistent samples with 4-Obs values of 1111, 2222, 3333, and 4444, respectively. Black points represent inconsistent samples. The red solid line shows the regression line between AveObs and L* − 4C*, with the correlation coefficient (r-value) shown in the upper-right corner.

The XY-axis scatter distribution of the AveObs versus L* − 4C* value is displayed in Figure 4. As the AveObs value increased, the L* − 4C* value decreased, with a correlation coefficient of −0.810. Among the consistent samples, it was difficult to distinguish the 1111 and 2222 samples because most of the L* − 4C* values were within the range of 30 to 70. Similarly, the 3333 and 4444 samples had overlapping distributions, with the majority of the L* − 4C* values less than 40. The L* − 4C* values of the 1111 (and 2222) versus 3333 (and 4444) samples were largely delineated around 35 (from 30 to 40) as a boundary interval. Notably, the 1111 and 2222 samples primarily had L* − 4C* values > 40, while the 3333 and 4444 samples mostly had L* − 4C* values < 30.

**Figure 5:**
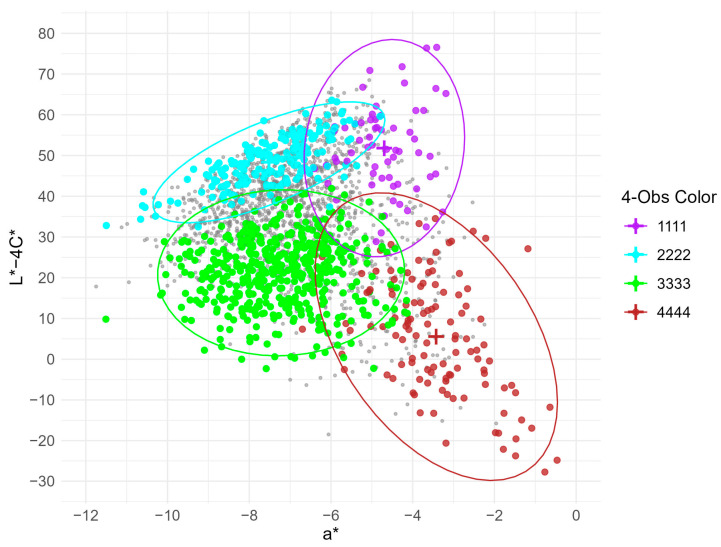
Scatter plot of a* and L* − 4C* values. Purple, blue, green, and brown points represent consistent samples with 4-Obs values of 1111, 2222, 3333, and 4444, respectively. The corresponding 95% density ellipses and centroids are depicted by matching-colored ellipses and crosses. Gray points represent inconsistent samples.

The XY-axis scatter distribution of the a* values versus the L* − 4C* values is displayed in Figure 5. As shown, the distributions of consistent samples (i.e., 1111, 2222, 3333, and 4444) were evidently different in the two-dimensional plot of a* and L* − 4C*. The majority of 1111 and 2222 samples had L* − 4C* values greater than 40, whereas most 3333 and 4444 samples had L* − 4C* values less than 30 (the same results are also demonstrated in Figure 4). Furthermore, the majority of the 1111 and 4444 samples had a* values greater than −5, whereas most of the 2222 and 3333 samples had a* values less than –6. These further results indicate that consistent samples can be mostly distinguished based on L* − 4C* with a* as auxiliary parameter. For the 1111, 2222, 3333, and 4444 samples, the classification thresholds of L* − 4C* and a* were >40 and >−5, >40 and <−6, <30 and <−6, and <30 and >−5, respectively.

## 4. Discussion

### 4.1. Batch Correction for Measurement of Eggshell Color

Generally, chickens that produce eggs with blue-green shells have not undergone long-term, highly intensive, systematic breeding as compared to commercial breeds producing eggs with brown and white shells, resulting in various shell colors within the population. Dalirsefat (2015) found that even when the major gene *SLCO1B3* controlling blue-eggshell was homozygous in blue-green eggshell populations, considerable variations in shell color still persisted [14]. Xu (2018) measured the shell colors of Changshun blue eggshell chickens across three groups (light green, dark green, and brown) and found that the Δa* values (mean ± SD) were −7.29 ± 2.27 for light green and −3.79 ± 2.39 for dark green groups, while the Δb* values were 4.70 ± 3.72 and 13.85 ± 3.85, respectively [15]. Chen and Wang (2022) reported that blue-green eggs from Lueyang black-bone chickens had a* and b* values of −1.7 ± 1.5 and 12.2 ± 2.6, respectively [27]. The degree of color variation in these studies generally exceeded or approximated those in the present study, indicating abundant diversity in the coloration of blue-green eggshells. Moreover, shell color variation exists even within the same breed across different laying periods. Yang et al. (2013) and Zhou et al. (2016) reported that shell color gradually became lighter from the early to late laying phases of Subei black-bone chickens and Lushi blue eggshell chickens [28,29]. In the present study, seven batches of eggs collected at three different time points (200, 250, and 300 days) from four generations (G5–G8 from 2020 to 2023) were included for analysis. Hence, systematic correction to the original data of shell color measurements across years and ages, which seemed necessary, was conducted with the ComBat function to specifically address batch effects and experimental timelines. After correction, the results showed substantially reduced CVs among the batches, confirming effective control of technical variability. Notably, visual shell color classification was retained as a genuine biological variation, although visual determination of shell color is subjective. So, in order to minimize observer subjectivity, standardized protocols were implemented, including fixed observation conditions, multiple independent observers, and unified classification criteria [16].

### 4.2. Machine Learning for Shell Color Classification

Zhao and Zhou (2019) utilized the fuzzy radial basis function NNET to predict egg freshness (i.e., Haugh unit) based on the hue, intensity, and saturation values of shell color, achieving accuracies of 95.175% for brown-shelled eggs and 97.525% for white-shelled eggs [30]. Mamiunah et al. (2018) utilized an SVM classifier to predict egg quality (e.g., color intensity) based on red, green, and blue values, reporting an accuracy of 80% in the categorization of three classes (dark brown, brown, and light brown) [31]. However, these studies relied on eggs from intensively selected breeds with uniform and distinct shell colors (brown or white), which contributed to the high classification performance. In contrast, blue-green eggs exhibit complex coloration due to variable contents and mixtures of protoporphyrin-IX and biliverdin [6,15], rendering classification more challenging than for eggs with brown or white shells. Wang et al. (2023) demonstrated that classification of blue-green eggs by four independent observers outperformed single-observer judgments, and the use of a single representative egg per hen outperformed classifying multiple eggs from the same hen [16]. Hence, the present study followed the classification methodology of Wang et al. (2023) [16], which included four consistent categories with 4-Obs values of 1111, 2222, 3333, and 4444, and other inconsistent categories (e.g., 1112, 1122, 1123, 1234), and 13 averaged visual shell color classifications (i.e., AveObs = 1.00, 1.25, 1.50, …, 4.00) derived from the mean classifications of the four observers. Among the four classical algorithms compared, LDA is a supervised dimensionality reduction method, maximizing inter-class variance, while minimizing intra-class variance, which is particularly effective for normally distributed data with equal covariance matrices, whereas RF is an ensemble algorithm using multiple decision trees with majority voting, offering robustness and suitability for high-dimensional data, SVM employs radial basis kernel functions for nonlinear classification via high-dimensional space mapping, and NNET mimics biological neural structures through multilayer perceptions, thereby optimizing weights via backpropagation [23]. The relatively poor performance of RF and NNET in this study can be attributed to the linear nature of relationships in measured L*a*b* values, which may not require the complex non-linear transformations that these models are designed to capture. In contrast, the results of the present study showed that the LDA model achieved the highest accuracy and efficiency, demonstrating the best comprehensive performance. This indicates that relatively simple algorithms (i.e., LDA) can satisfactorily predict eggshell color categories from the L*, a*, and b* values, likely because shell color classification naturally represents an ordinal multiclass classification with gradual transitions from category 1 (Light) to 4 (Olive). For consistent samples, the accuracy of LDA exceeded 94%, suggesting capability for accurate classification of standard four categories. Regarding all samples, the accuracy of LDA approached 45%, indicating challenges to classification of controversial categories (i.e., inconsistent samples), yet achieved a substantial improvement as compared to the theoretical random probability of only 7.69% (1/13 classifications of AveObs).

### 4.3. Shell Color Indices for Blue-green Eggs

The formula C* (=$\sqrt{{a*}^{2}+{b*}^{2}})\;$incorporates only two parameters: the red-green chromaticity a* and the yellow-blue chromaticity b*. Moreno et al. (2004) and Morales et al. (2010) applied the C* value to study blue-shelled eggs of the pied flycatcher in natural environments [22,32], while Lukanov et al. (2015) used it to compare colors of white, pink, blue-green, and brown shells of chicken eggs [33]. The SCI (= L* − a* − b*) was gradually developed by Lohmann Breeders GmbH (Cuxhaven, Germany) for selection of eggs with brown shells. The formula L* − a* − b* includes all three parameters (L*, a*, and b*) without weighting coefficients. The lower SCI value of brown-shelled eggs indicates a darker eggshell color, providing a continuous variable scale for selection of dark-brown eggs [12]. In this study, the classification results from the LDA algorithm supported the construction of other simplified color indices because the LD1 function, composed of L*, a*, and b* with proportional coefficients, accounted for over 78% of the model contribution. Following the dimensionality reduction principle of LDA, two simplified equations were developed and optimized based in the indices C* and SCI: L* − kb*, which eliminates the a* value, as compared to SCI, while introducing a coefficient for b*, allowing a* to serve as an independent dimension for classification. The equation L* − kC* incorporates both a coefficient for C* and the L* parameter, thereby encompassing all three parameters (i.e., L*, a*, and b*) like SCI. After determining the optimal coefficient k, both simplified formulas (L* − 2b* and L* − 4C*) demonstrated higher correlations with shell color classification than C* and SCI. Especially, the correlation between L* − 4C* and AveObs showed statistical significance as compared to SCI (*p* < 0.05), indicating the potential to streamline shell color categorization for blue-green eggs in practical applications. For example, consumers can broadly distinguish light-to-blue-shelled (L* − 4C* > 40) and green-to-olive-shelled (L* − 4C* < 30) eggs, according to the results of the present study. If considering a* (the major component in the LD2 function of LDA model) additionally, consumers can further distinguish light- and olive-shelled (a* > −5) from blue- and green-shelled eggs (a* < −6). Future research on shell color indices and eggshell pigment content is expected to provide complementary scientific support.

## 5. Conclusions

Among the four machine learning algorithms evaluated, the LDA model demonstrated optimal comprehensive performance in predicting color classification of blue-green eggshells using measured L*a*b* values, with the highest accuracy and efficiency. The methodology of simplifying complex models to simple formulas led to the development of the L* − 4C* index, which exhibited the strongest correlation (r = −0.810) with visual classification as compared to L* − 2b* and existing indices (C* and SCI). Furthermore, L* − 4C* provides a threshold to distinguish light-to-blue shells (>40) and green-to-olive shells (<30), offering a practical solution for color grading of blue-green egg production.

## Figures and Tables

**Table 1:** Sampling details of the experimental eggs.

Generation	G5	G6		G7		G8	
Days of Age	250 d	200 d	300 d	200 d	300 d	200 d	300 d
Batch	1	2	3	4	5	6	7
Egg number	484	458	398	320	196	242	176

**Table 2:** Statistical details of 4-Obs and AveObs.

No.	AveObs	4-Obs	N (Cons.)	N (Incons.)	N (All)	% of Total
1	1.00	1111 ^1^	63	0	63	2.8
2	1.25	1112	0	29	29	1.3
3	1.50	1113, 1122	0	69	69	3.0
4	1.75	1114, 1123, 1222	0	135	135	5.9
5	2.00	1124, 1133, 1223, 2123, 2222 ^1^	199	80	279	12.3
6	2.25	1134, 1224, 1233, 2223	0	260	260	11.4
7	2.50	1144, 1234, 1333, 2233	0	287	287	12.6
8	2.75	1334, 2234, 2333	0	337	337	14.8
9	3.00	1344, 2334, 3333 ^1^	484	9	493	21.7
10	3.25	1444, 2344, 3334	0	95	95	4.2
11	3.50	2444, 3344	0	59	59	2.6
12	3.75	3444	0	54	54	2.4
13	4.00	4444 ^1^	114	0	114	5.0

^1^ 1111, 2222, 3333, and 4444 mean that all four observers agreed on the same visual shell color classification, also referring to the “consistent samples”. “No.” indicates the row number. “Cons.” and “Incons.” are consistent and inconsistent samples, respectively.

**Table 3:** Coefficient of variation (CV) of the L*, a*, and b* values of seven batches before and after correction (corr.).

	Before Corr. CV (%)	After Corr. CV (%)	Reduction Rate (%)
L*	1.55	0.35	77.4
a*	4.42	0.71	83.9
b*	8.40	2.70	67.9

**Table 4:** Statistical summary of the L*, a*, b*, and AveObs values after correction.

	L*	a*	b*
*Mean ± SD, CV%*	*81.82 ± 4.24, 5.19%*	*−6.74 ± 1.65, 24.51%*	*9.76 ± 3.99, 40.84%*
a*	−0.088		
b*	−0.722	0.451	
AveObs	−0.713	0.218	0.771

Italic text denotes the mean and standard deviation (SD) of the L*, a*, and b* values of all samples, with coefficient of variation (CV) behind the comma. Lower triangle (normal font) presents correlation coefficients between the L*, a*, b*, and AveObs values.

**Table 5:** Classification performance of the four models in three data scenarios.

Sample	Model	Accuracy	F1-Score	Kappa	Noise Sensitivity	Prediction Efficiency
All	LDA	0.450	0.391	0.357	0.284	172,803
	RF	0.395	0.320	0.305	0.461	848
	SVM	0.439	0.472	0.341	0.287	2352
	NNET	0.408	0.390	0.306	0.261	157,423
Cons.	LDA	0.941	0.914	0.903	0.018	129,695
	RF	0.934	0.889	0.889	0.038	37,059
	SVM	0.941	0.911	0.902	0.017	23,885
	NNET	0.934	0.896	0.889	0.024	72,194
Incons.	LDA	0.448	0.388	0.335	0.098	140,334
	RF	0.348	0.343	0.220	0.332	14,681
	SVM	0.436	0.482	0.314	0.095	3580
	NNET	0.386	0.341	0.261	0.117	115,515

“Cons.” and “Incons.” indicate consistent and inconsistent samples, respectively.

**Table 6:** Correlation coefficients (r-values) between shell color indices and AveObs, along with *p*-values for difference tests.

	SCI	C*	L* − 2b*	L* − 4C*
*r*	*−0.782*	*0.797*	*−0.803*	*−0.810*
SCI		0.179	0.057	0.010 *
C*			0.574	0.216
L* − 2b*				0.500

Italic text denotes the correlation coefficient between the shell color index and AveObs value. Upper triangle (normal font) presents *p*-values for pairwise comparisons of these correlations, with asterisk (*) indicating statistically significant differences (*p* < 0.05).

## Data Availability

The original contributions presented in this study are included in the article/Supplementary Material. Further inquiries can be directed to the corresponding author.

## Supplementary Materials

The following supporting information can be downloaded at: https://www.mdpi.com/article/10.3390/foods14173027/s1, Table S1: Whole dataset of eggshell color; Table S2: Parameter specifications of four algorithms; Table S3: Precision, recall, and training efficiency of four models in three data scenarios; Table S4: Confusion matrix of four models in three data scenarios; File S1: Detailed R code for batch correction.
