# Supplementary material for: Improved Shell Color Index for Chicken Eggs with Blue-green Shells Based on Machine Learning Analysis
Source: Foods. 2025 Aug 29;14(17):3027. doi: 10.3390/foods14173027 (PMC12428762; doi:10.3390/foods14173027)
Supplement: Supplementary file 1 [file foods-14-03027-s001.zip › Supplementary File S1.pdf]

```r
# ===== Batch Correction Code =====
# 1. Load required packages
library(sva)

# 2. Prepare data
# Assuming data is loaded as 'data', containing columns: batch, L_value (i.e., L*), a_value (i.e., a*),
b_value (i.e., b*), 4-Obs, visual_score (i.e., AveObs)
lab_matrix <- as.matrix(data[,c("L_value", "a_value", "b_value")])
batch <- as.factor(data$batch)

# 3. Perform ComBat batch correction
combat_result <- ComBat(dat = t(lab_matrix),
                        batch = batch,
                        mod = model.matrix(~visual_score, data = data),
                        par.prior = TRUE,
                        prior.plots = FALSE)

# 4. Transform corrected data back to original format
corrected_lab_matrix <- t(combat_result)

# 5. Update dataset
data$L_value_corrected <- corrected_lab_matrix[,1]
data$a_value_corrected <- corrected_lab_matrix[,2]
data$b_value_corrected <- corrected_lab_matrix[,3]

# 6. Validate batch correction effectiveness
# Calculate coefficient of variation (CV) before correction
cv_before <- sapply(c("L_value", "a_value", "b_value"), function(var) {
  sd(aggregate(data[[var]], by=list(data$batch), mean)$x) /
    mean(data[[var]]) * 100
})

# Calculate coefficient of variation (CV) after correction
cv_after <- sapply(c("L_value_corrected", "a_value_corrected", "b_value_corrected"), function(var)
{
  sd(aggregate(data[[var]], by=list(data$batch), mean)$x) /
    mean(data[[var]]) * 100
})

# Output results
print("Coefficient of variation before correction (%):")
print(round(cv_before, 2))
print("Coefficient of variation after correction (%):")
print(round(cv_after, 2))
```
